# A Novel Mutation-*BRCA1* Associated Hereditary Haplotype of Intragenic Markers of *BRCA1 *Gene in a Family with History of Breast Cancer

**DOI:** 10.31557/APJCP.2019.20.2.611

**Published:** 2019

**Authors:** Seyed Mohsen Miresmaeili, Fatemeh Jafari

**Affiliations:** *Department of Biology, Science and Arts University, Yazd, Iran. *

**Keywords:** Breast cancer, intronic mutation, haplotype, BRCA1 gene

## Abstract

**Background:**

Breast cancer is the most common cancer diagnosed among women, Tumor suppressor genes such as* BRCA1* involved in cell cycle control and repairing of DNA damage.* BRCA1* is a risk factor gene that alteration in its protein cause in susceptibility to breast or ovarian cancer. Short tandem repeat (STR) polymorphism is linked to some disease.

**Objective:**

The aim of this study was screening a new mutation in patients with familial breast cancer.

**Materials and Methods:**

In this study, 200 women with breast cancer were participated. Among the patients, 40 women suffer from familial breast cancer. After DNA extraction from peripheral blood samples, Exons 16 to 23 of* BRCA1* gene directly analyzed in SSCP gel electrophoresis followed by direct sequencing.

**Results:**

After direct sequencing, a new mutation was detected in intron 17 of* BRCA1* gene. Three patients of one family have a germ line intronic mutation in the* BRCA1* gene (IVS17-27delA). Also, this mutation in this family is linked to a haplotype of intragenic short tandem repeat (STR) in the* BRCA1* gene.

**Conclusion:**

By Screening of gene mutations can be found association of mutation and incidence of disease. Also, studying the mutation in families and finding specific hereditary patterns in that family can be effective in prognosis of disease in other family members.

## Introduction

Breast cancer is the most common cancer in women worldwide. Tumor suppressor genes involved in cell cycle control and repairing of DNA damage. *BRCA1* (Breast Cancer Type 1 Susceptibility gene) is a risk factor gene that alteration in its protein cause in susceptibility to breast or ovarian cancer. The most common malignancy in women is breast cancer. About 12% of all new cancer cases and 25% of all cancers in women is breast cancer (World Cancer Research Fund International, 2017). *BRCA1* is a tumor suppressor gene that participate in DNA damage repair in normal cells(Miki et al., 1994). Familial history is a risk factor for breast cancer involved in more than 20% of all breast cancer cases. More than 5% of all breast cancer have been inherited as an autosomal pattern (Selvi, 2015). *BRCA1* protein consist of 1863 amino acids. Functional structure of *BRCA1* protein involve three major domains including an ubiqitin ligase domain (RING domain) at the N-terminus (Morris et al., 2006), nuclear localization signal domain (NLS) in the middle (Zhang et al., 1998) and BRCT (*BRCA1* C-terminal) domain at the C-terminus (Huyton et al., 2000). BRCT domain encoded by exon16-23 that involve in DNA damage repair and cell cycle control and interaction with other proteins (Clark et al., 2012). Therefore, researcher focus on functional domains of *BRCA1* gene. Inherited mutations in the *BRCA1* gene are responsible for the major hereditary breast cancer cases. Deficiency in *BRCA1* gene is considered in high risk family with breast cancer. Several mutations have been reported associated with breast cancer in Iranian patients (Desantis et al., 2014; Forat-yazdi et al., 2015). In some disease, intragenic marker polymorphism be used as an explorer tool for finding patients carrying mutation (Ali et al., 2007; Miresmaeili et al., 2016; Nowacka-Zawisza et al., 2008; Osorio et al., 2003). STR markers of a gene are good evidence for following of mutation in a family or population. Three intragenic STR markers (D17S855, D17S1322 and D17S1323) localized in *BRCA1* gene. Therefore, screening of mutation in high risk genes may help in medical management of patients and their families Genetic (Drost and Jonkers, 2014; Kobayashi et al., 2013). Our main goal was to screening of new mutation in BRCT domain of *BRCA1* gene in patients with familial breast cancer.

## Materials and Methods

Experimental study was carried out in Science and Arts University, Yazd, Iran. The study protocol was approved by the Ethics Committee of Shahid Sadoughi University of Medical Sciences, Yazd, Iran. Written informed consent was obtained from all participants. Patients recruited were diagnosed with breast cancer. In a prospective study A total of 200 breast cancer patients, the age at diagnosis of first primary was under 51 years (range: 36–51 years), were admitted to several hospital in Yazd, Iran. Among the patients, 40 women suffer from familial breast cancer that have participated in our study. Peripheral Blood samples were collected in EDTA. DNA extraction procedure, PCR conditions, SSCP conditions and direct sequencing previously reported(Miresmaeili et al., 2016). Primers used have been chosen from Breast Cancer Information Core Database (BIC) or designed by Sci-Ed Software (Table 1). DNA Sequences were compared against reference sequence (NG_005905.2) using chromas (v.2.2) and NCBI softwers.

**Table 1:** Primer Sequences for BRCT Domain of *BRCA1*

Exon No.	Forward primer	Reverse primer	Annealing (^o^C)	Product size (bp)	Ref.
16	5′ AATTCTTAACAGAGACCAGAAC	5′AAAACTCTTTCCAGAATGTTGT	58	450	Friedman LS, 1994
17	5′ GTGTAGAACGTGCAGGATTG	5′ TCGCCTCATGTGGTTTTA	55	263	BIC database
18	5′ GGCTCTTTAGCTTCTTAGGAC	5′ CTCAGACTCAAGCATCAGC	55	260	BIC database
19	5′ CTGTCAATCTTCCTGTGCTC	5′CATTGTTAAGGAAAGTGGTGC	58	249	Friedman LS , 1994
20	5′ TATGACGTGTCTGCTCCAC	5′ AGTCTTACAAAATGAAGCGG	55	258	Sci-Ed Software
21	5′ GACATTGGACTGCTTGTC	5′GTAGAGAAATAGAATAGCCTCT	58	366	Sci-Ed Software
22	5′ CCCATTGAGAGGTCTTGCT	5′ GACATTTTAGCCATTCATTCA	60	296	Sci-Ed Software
23	5′ AAAATGATGAAGTGACAGTTC	5′ GACATTTTAGCCATTCATTCA	55	267	BIC database

**Figure 1:**
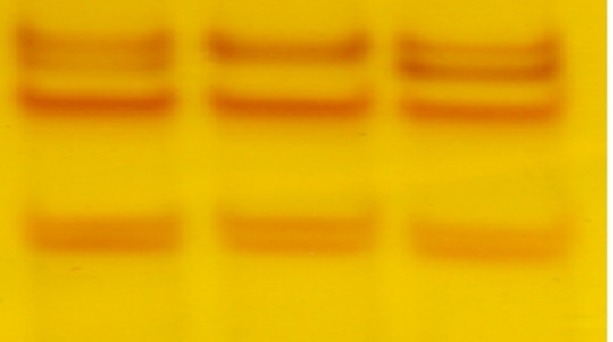
Single Strand Conformational Polymorphism Showing Mobility Shift with Patient 2

**Figure 2:**
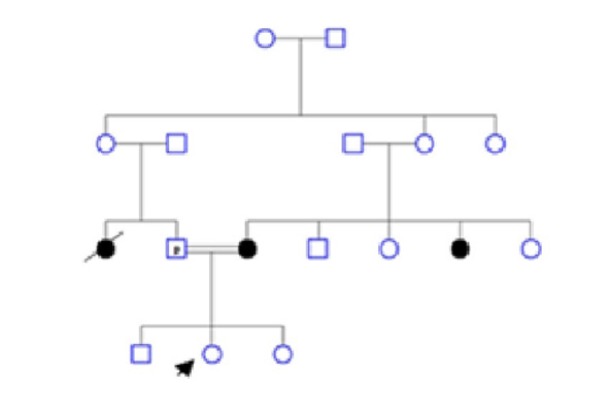
The Novel Mutation in Intron 17 of *BRCA1*, IVS17-21delA

**Figure 3:**
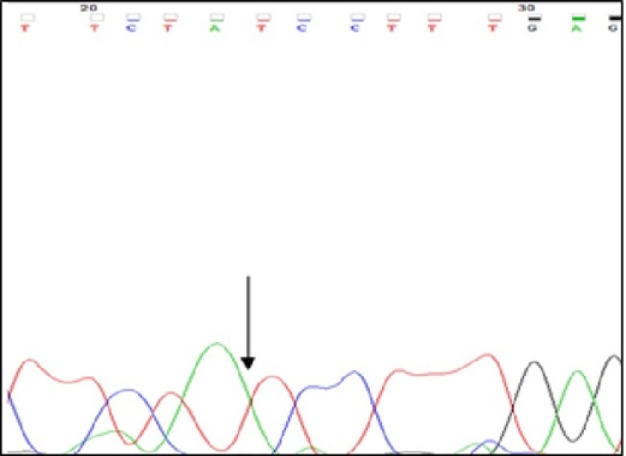
Pedigree of the Family Presenting Novel Mutation. Circles with solid dark color indicate the affected individuals. P, prostate cancer; arrow, the healthy daughter

## Results

The BRCT domain in *BRCA1* gene of 40 Iranian patients with a family history of breast cancer was analyzed. The entire coding region and intronic sequences flanking each exons involved BRCT domain (exon 16-23) were analyzed using single-stranded conformational polymorphism (PCR-SSCP) followed by direct DNA sequencing. In comparison with normal healthy women, the SSCP band patterns in one family cancerous members were different in PCR product for exon 17 (Figure 1). Haplotype analyses of three intragenic STR marker in *BRCA1* gene in participants were carried out by fragment analysis previously (Miresmaeili et al., 2016). After direct sequencing, we found a novel intronic mutation (IVS17-27delA) in *BRCA1* gene which was inherited in the cancerous members of one family (Figure 2). 

## Discussion

A family with history of breast cancer displays a novel intronic mutation in cancerous members and its inheritance linked to a common haplotype. Pedigree of family is shown in Figure 3. All breast cancer patients in family undergoing surgery. This nucleotide deletion (IVS17-21delA) not finds in another patients or healthy persons. Three patients of one family display this deletion but it was identified in healthy daughter (20 years old) of family. In the other study, there was a new mutation in cancerous members of one family with breast and ovarian cancer (Calò et al., 2006; Cortesi et al., 2003). In the other hands, Intronic mutations approved and considered for incidence of disease or in Scarff-Bloom-Richardson (SBR) grading of breast cancer (Anczuków et al., 2012; Dancourt et al., 2006; Memni et al., 2016; Mundhofir et al., 2016; Pupo et al., 2017). Therefore, screening of this new intronic mutation in other familial cancer is important for managing of incidence and prognosis of breast cancer. To investigate whether the recurrence of this mutation is linked to STR markers, haplotype analyses were performed with 3 intragenic polymorphic markers (D17S855, D17S1322 and D17S1323) located in intron 20, intron 19 and intron 12, respectively. Based on allele, a common haplotype linked to IVS17-21delA mutation. Common haplotype in involved family was 153,121,159 for D17S855, D17S1323 and D17S1322 respectively, unaffected daughter has not shown any clinical phenotype. She represent the same family haplotype. Maybe, she will be diagnosed with breast cancer in the future. Previous study in woman suffer from breast cancer suggested that One haplotype of the ESR2 gene was associated with breast cancer risk(Cox et al., 2008). Also, analysis in TP53 gene identified a haplotype that associated with a significantly decreased breast cancer risk(Vymetalkova et al., 2015). Our Identified variation (IVS17-21delA), probably, associated with an increased risk of breast cancer. But further studies will be reveal relation of this mutation with breast cancer. In confirmation of this study, allele variation in STR marker in RAD51 gene associated with breast cancer (Nowacka-Zawisza et al., 2008). Also, in other studies, researchers have been found alternative haplotype association with various cancer (Huang et al., 2007; Ozolina et al., 2009; Panczyk et al., 2009). Screening of mutations is the best way to direction of research for treatment of disease, it can be useful to finding association of mutation and incidence of disease. Also, studying the mutation in families and finding specific hereditary patterns in that family can be effective in prognosis of disease in other family members.

## References

[B1] Ali AB, Iau PT, Putti TC, Sng JH (2007). BRCA1 disease-associated haplotypes in Singapore Malay women with early-onset breast/ovarian cancer. Breast Cancer Res Treat.

[B2] Anczuków O, Buisson M, Léońe M (2012). BRCA2 deep intronic mutation causing activation of a cryptic exon: Opening toward a new preventive therapeutic strategy. Clin Cancer Res.

[B3] Calò V, Agnese V, Gargano G (2006). A new germline mutation in BRCA1 gene in a sicilian family with ovarian cancer. Breast Cancer Res Treat.

[B4] Clark SL, Rodriguez AM, Snyder RR, Hankins GDV, Boehning D (2012). Structure-function of the tumor suppressor BRCA1. Comput Struct Biotechnol J.

[B5] Cortesi L, Turchetti D, Bertoni C (2003). Italian family with two independent mutations: 3358T/A in BRCA1 and 8756delA in BRCA2 genes. Eur J Hum Genet.

[B6] Cox DG, Bretsky P, Kraft P (2008). Haplotypes of the estrogen receptor beta gene and breast cancer risk. Int J Cancer.

[B7] Dancourt J, Vuillaumier-Barrot S, De Baulny HO (2006). A new intronic mutation in the DPM1 gene is associated with a milder form of CDG Ie in two French siblings. Pediatr Res.

[B8] Desantis C, Ma J, Bryan L, Jemal A (2014). Breast cancer statistics 2013. CA Cancer J Clin.

[B9] Drost R, Jonkers J (2014). Opportunities and hurdles in the treatment of BRCA1-related breast cancer. Oncogene.

[B10] Forat-yazdi M, Neamatzadeh H, Hasan M (2015). BRCA1 and BRCA2 common mutations in Iranian breast cancer patients: a meta analysis BRCA1 and BRCA2 common mutations in Iranian breast cancer patients: a meta analysis. Asian Pac J Cancer Prev.

[B11] Huang YC, Chen M, Lin MW (2007). CYP19 TCT Tri-Nucleotide Del/Del genotype is a susceptibility marker for prostate cancer in a Taiwanese population. Urology.

[B12] Huyton T, Bates PA, Zhang X, Sternberg MJE, Freemont PS (2000). The BRCA1 C-terminal domain: structure and function. Mutat Res Repair.

[B13] Kobayashi H, Ohno S, Sasaki Y, Matsuura M (2013). Hereditary breast and ovarian cancer susceptibility genes (Review). Oncol Rep.

[B14] Memni H, Macherki Y, Klayech Z 2016), E-cadherin genetic variants predict survival outcome in breast cancer patients. J Transl Med.

[B15] Miki Y, Swensen J, Shattuck-Eidens D (1994). A strong candidate for the breast and ovarian cancer susceptibility gene BRCA1. Science.

[B16] Miresmaeili SM, Mohammad D, Tamandani K (2016). Haplotype analysis of BRCA1 intragenic markers in Iranian patients with familial breast and ovarian cancer. Int J Reprod BioMed.

[B17] Morris JR, Pangon L, Boutell C (2006). Genetic analysis of BRCA1 ubiquitin ligase activity and its relationship to breast cancer susceptibility. Hum Mol Genet.

[B18] Mundhofir FE, Wulandari CE, Prajoko YW, Winarni TI (2016). BRCA1 gene mutation screening for the hereditary breast and/or ovarian cancer syndrome in breast cancer cases: a First high resolution DNA melting analysis in Indonesia. Asian Pac J Cancer Prev.

[B19] Nowacka-Zawisza M, Brys M, Romanowicz-Makowska H, Kulig A, Krajewska WM (2008). Dinucleotide repeat polymorphisms of RAD51 BRCA1BRCA2 gene regions in breast cancer. Pathol Int.

[B20] Osorio A, de la Hoya M, Rodríguez-López R (2003). Over-representation of two specific haplotypes among chromosomes harbouring BRCA1 mutations. Eur J Hum Genet.

[B21] Ozolina S, Sinicka O, Jankevics E (2009). The 4154delA mutation carriers in the BRCA1 gene share a common ancestry. Fam Cancer.

[B22] Panczyk M, Balcerczak E, Piaskowski S (2009). ABCB1 gene polymorphisms and haplotype analysis in colorectal cancer. Int J Colorectal Dis.

[B23] Pupo GM, Boyd SC, Fung C (2017). Clinical significance of intronic variants in BRAF inhibitor resistant melanomas with altered BRAF transcript splicing Biomark. Res.

[B24] Selvi R (2015). Hereditary breast cancer in: Breast deasise: imaging and clinical management.

[B25] Vymetalkova V, Soucek P, Kunicka T (2015). Genotype and haplotype analyses of TP53 gene in breast cancer patients: Association with risk and clinical outcomes. PLoS One.

[B26] World Cancer Research Fund International (2017). Breast cancer statistics.

[B27] Zhang H, Somasundaram K, Peng Y (1998). BRCA1 physically associates with p53 and stimulates its transcriptional activity. Oncogene.

